# Supplementary material for: Study of the Interface between Wild Bird Populations and Poultry and Their Potential Role in the Spread of Avian Influenza
Source: Microorganisms. 2023 Oct 21;11(10):2601. doi: 10.3390/microorganisms11102601 (PMC10609042; doi:10.3390/microorganisms11102601)
Supplement: Supplementary file 1 [file microorganisms-11-02601-s001.zip › SM Figures.pdf]

microorganisms

MDPI

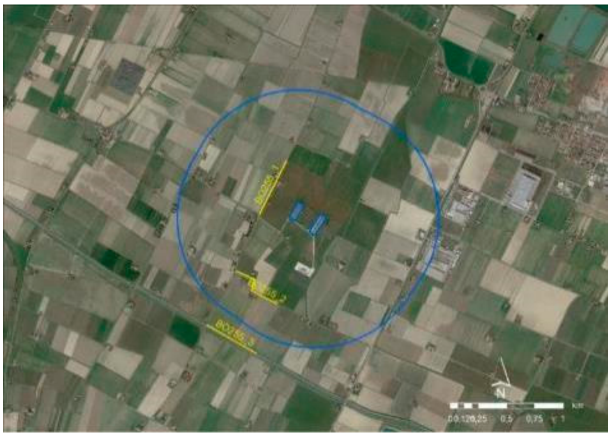

Farm BO-01

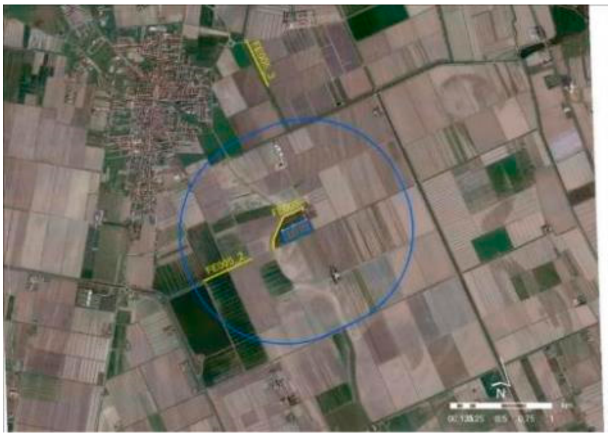

Farm FE-01

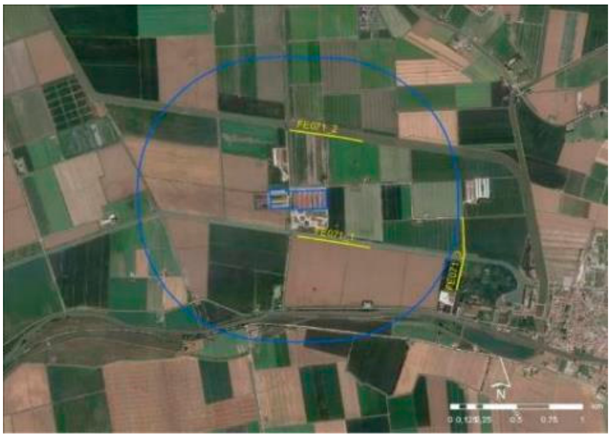

Farm FE-02

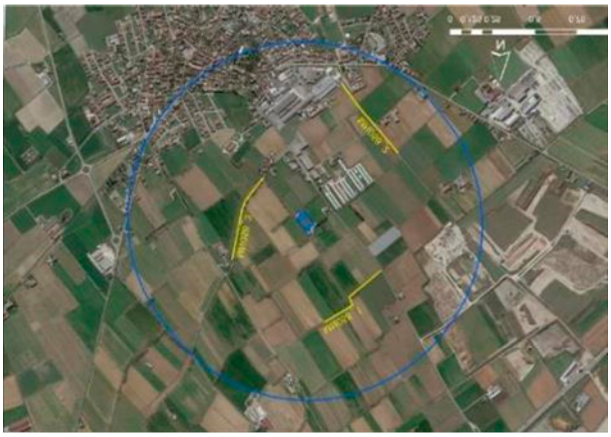

Farm MN-01

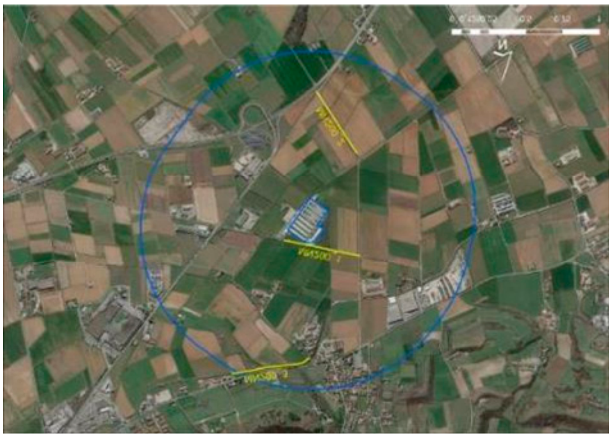

Farm MN-02

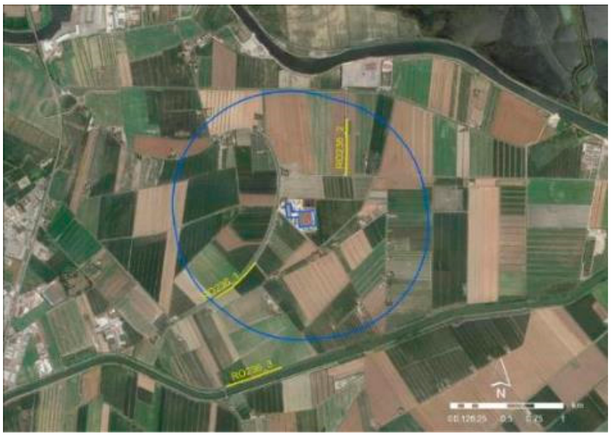

Farm RO-01

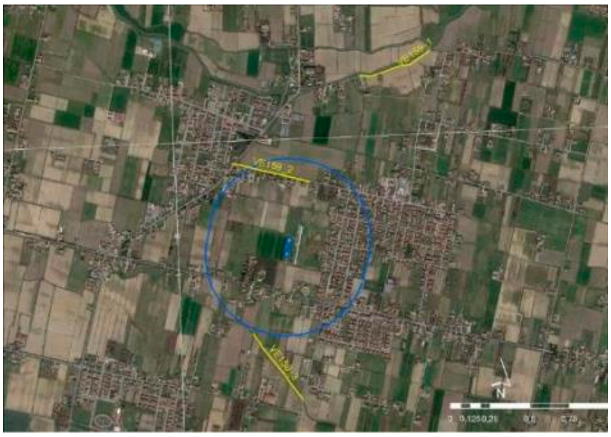

Farm VE-01

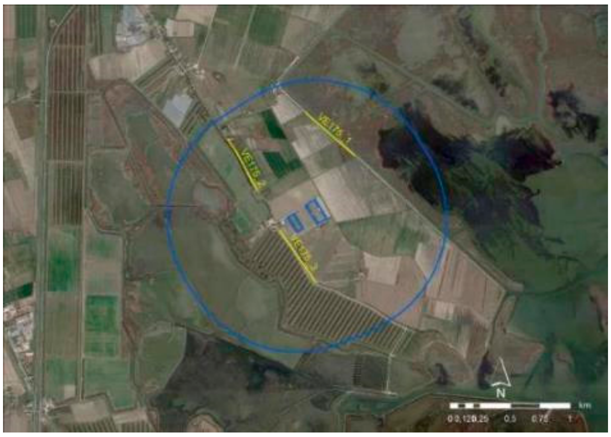

Farm VE-02

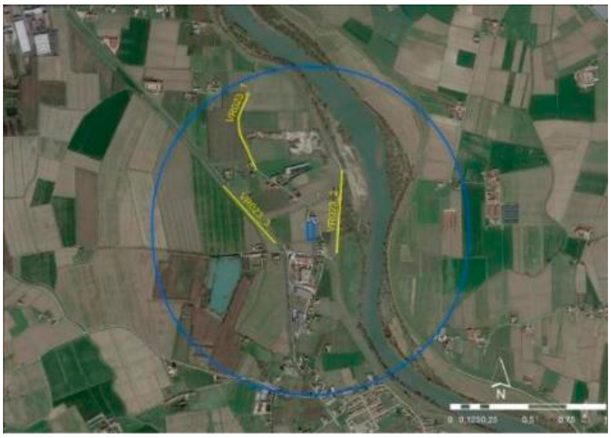

Farm VR-01

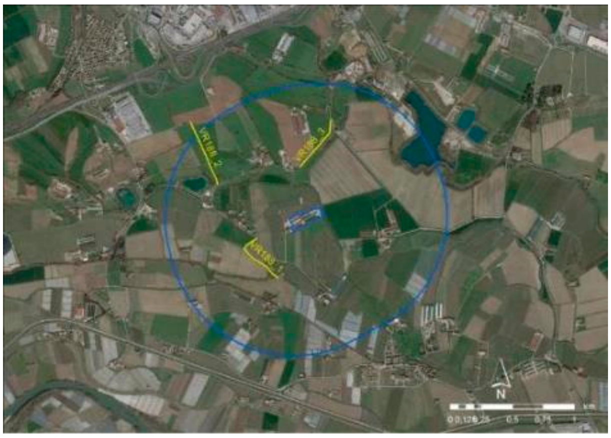

Farm VR-02

Figure S1. Domestic poultry farms included in the study. Yellow lines: transects used for ornithocenosis activities; blue circles: 1 km radius buffers around the farms.

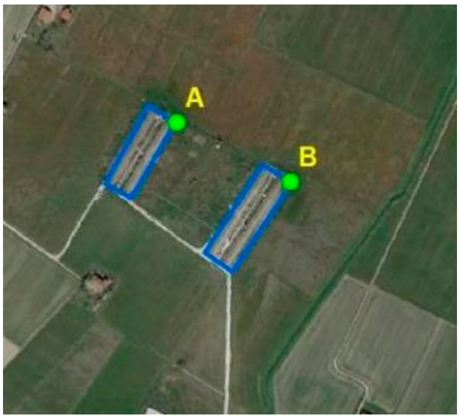

Farm BO-01

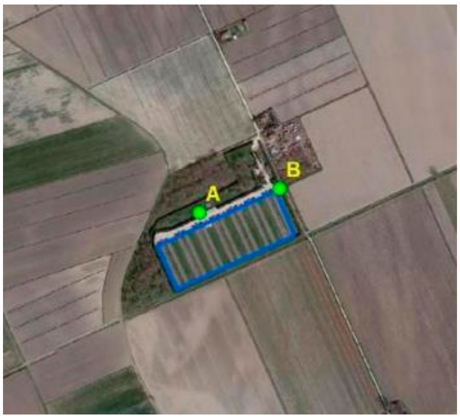

Farm FE-01

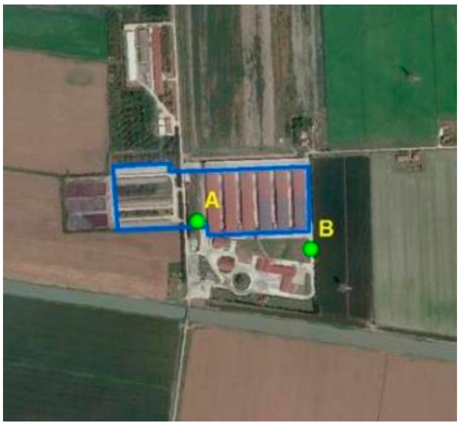

Farm FE-02

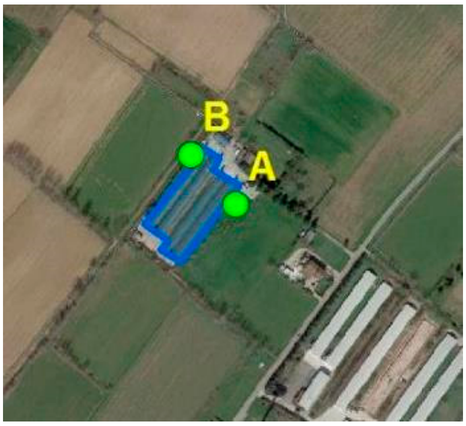

Farm MN-01

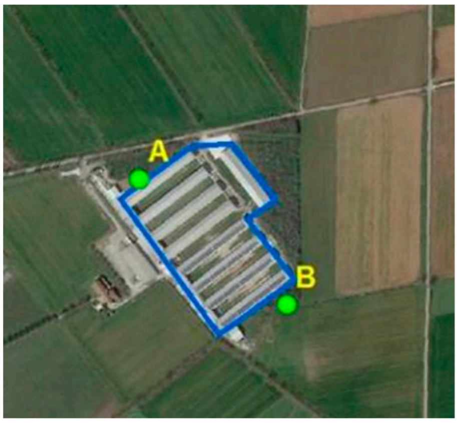

Farm MN-02

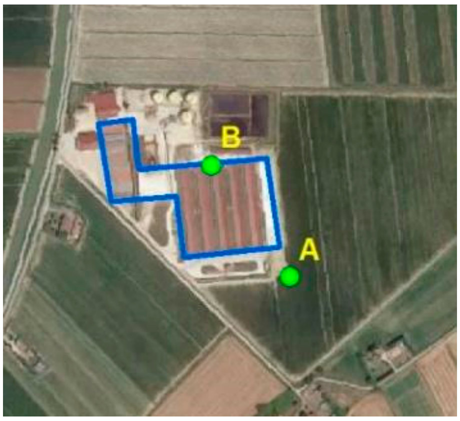

Farm RO-01

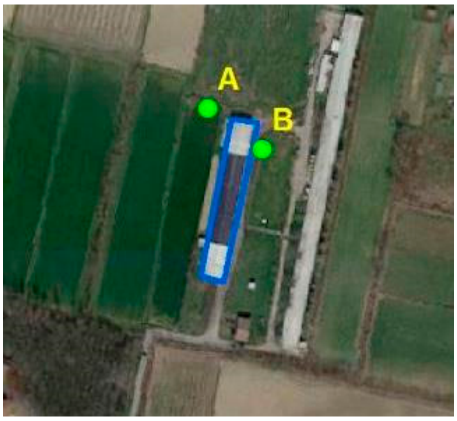

Farm VE-01

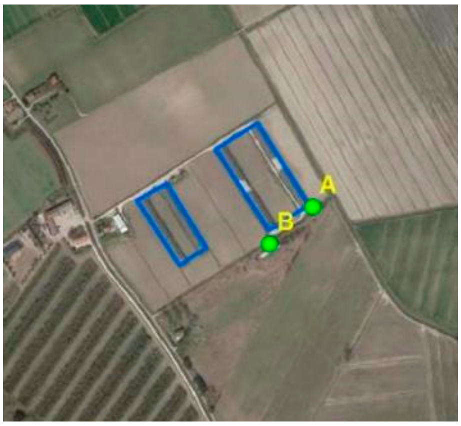

Farm VE-02

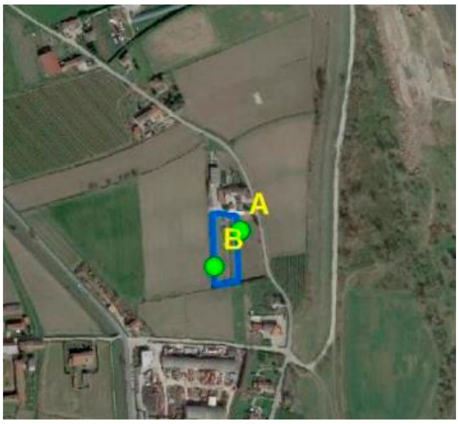

Farm VR-01

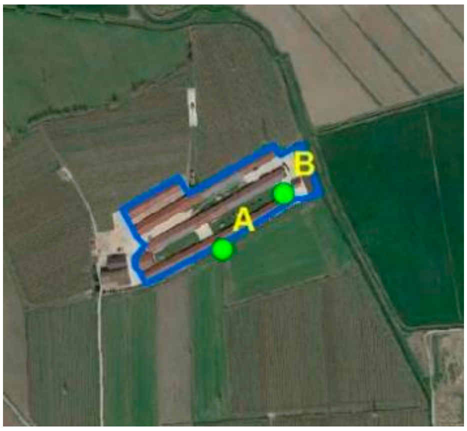

Farm VR-02

Figure S2. Location of the two camera traps (A and B) within the farms included in the study. Blue perimeters: farming area boundaries.

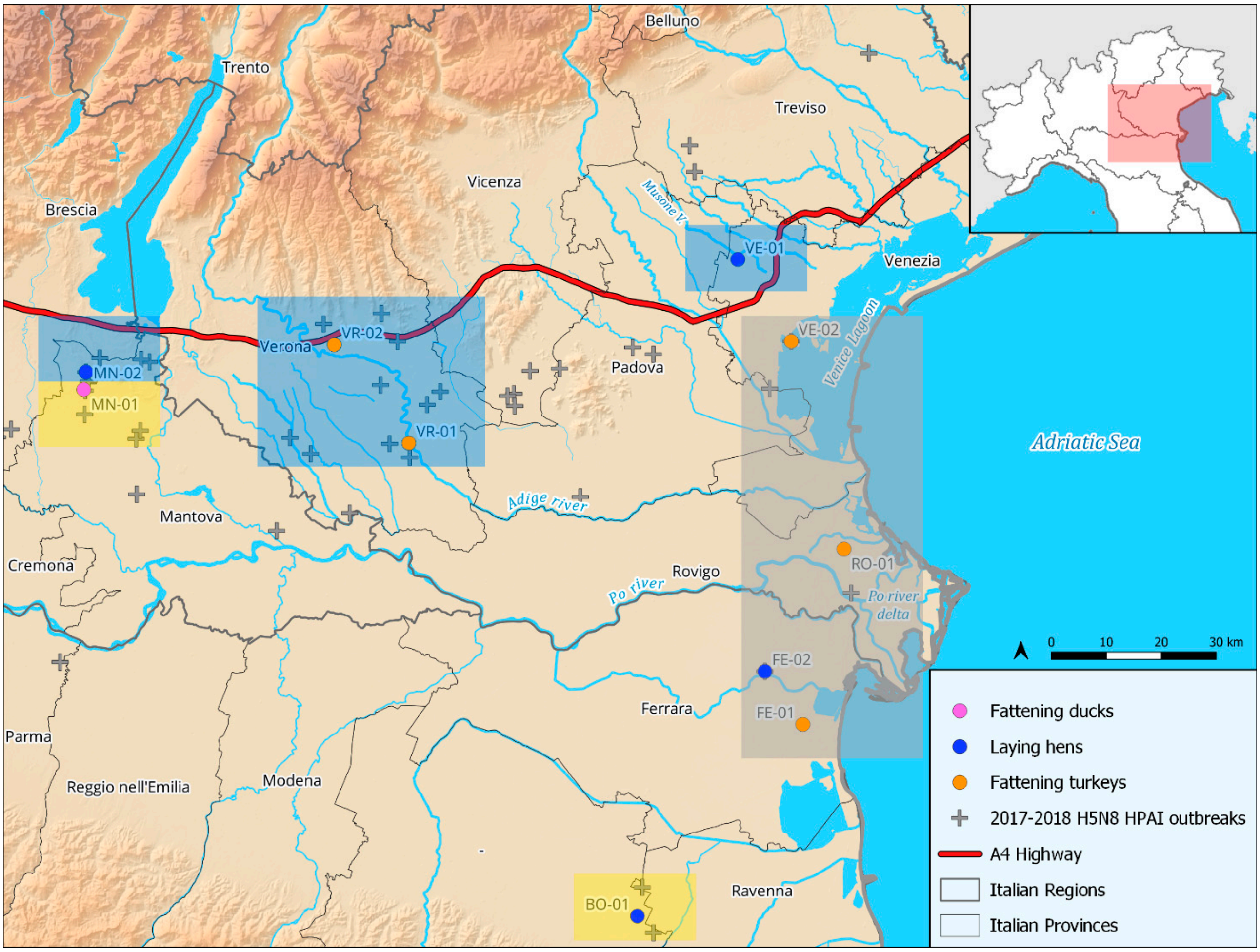

Figure S3. Geographic location of clusters detected through dendrogram and nMDS analysis. Grey area: Cluster 1; yellow areas: Cluster 2; blue areas: Cluster 3.

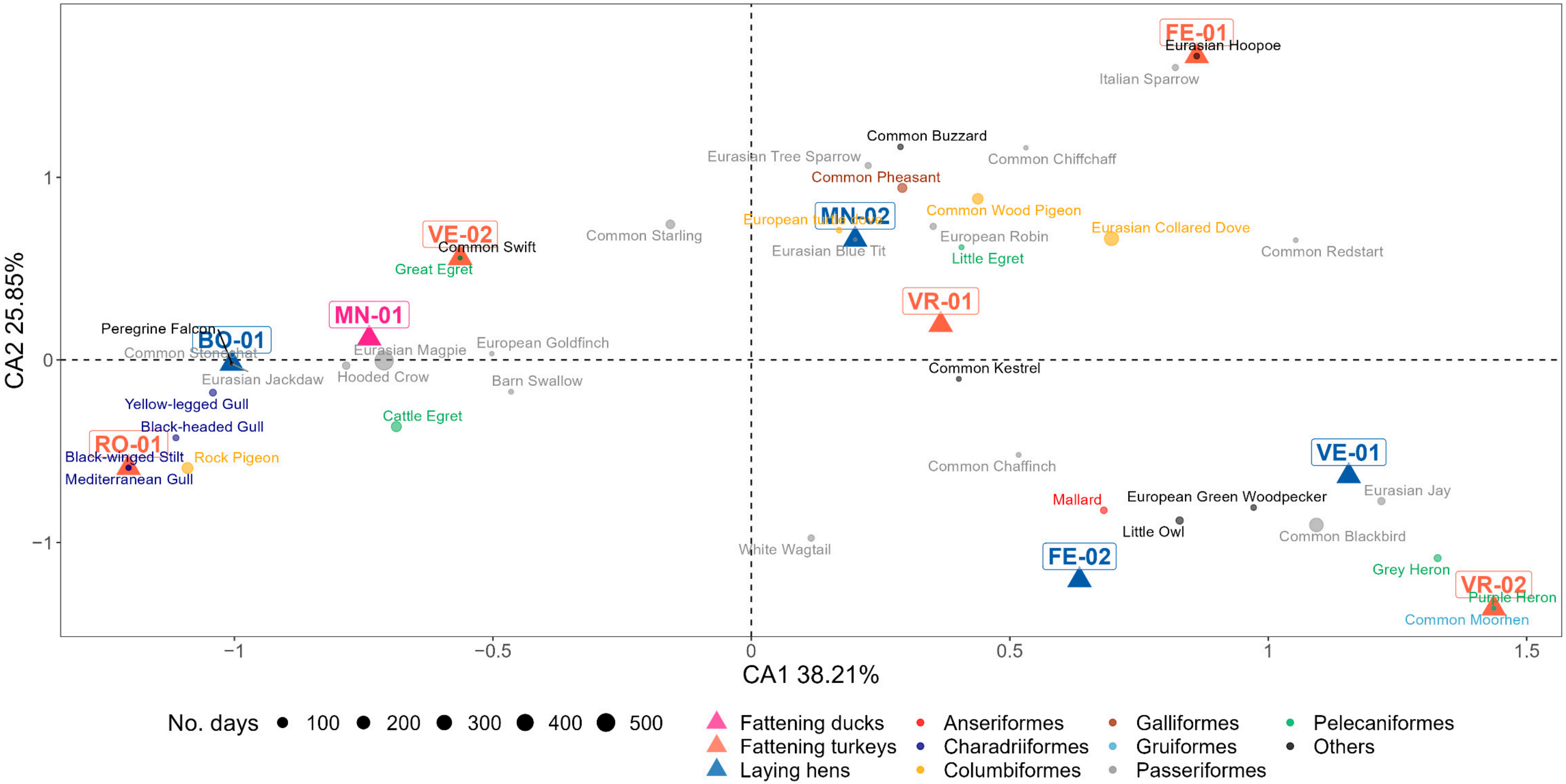

Figure S4. Correspondence analysis plot. Association between sites and camera-trapped species. The species are colored according to their Order. CA plot points' position reveals associations based on their angle and distance from the axes origin: same direction for positive, opposite for negative associations; shorter distances for even species distribution, longer for unequal abundances.
