# Supplementary material for: Study of the Interface between Wild Bird Populations and Poultry and Their Potential Role in the Spread of Avian Influenza
Source: Microorganisms. 2023 Oct 21;11(10):2601. doi: 10.3390/microorganisms11102601 (PMC10609042; doi:10.3390/microorganisms11102601)
Supplement: Supplementary file 1 [file microorganisms-11-02601-s001.zip › SM Tables.pdf]

microorganisms

Table S1. Wild birds contacted via ornithological transects. For each wild bird species, Order, scientific name, common name, total number of encountered animals belonging to that species, and percentage of the total encountered animals are reported. The last column, 'Target AI', identifies the wild bird species contacted in this study that belong to the list of 50 target species for passive surveillance established by EFSA (More et al., 2017).

Order	Scientific name	Common name	No. birds	Percentage	Target AI
Columbiformes	*Columba livia forma domestica*	Rock Pigeon	5890	11.16%	
Columbiformes	*Columba palumbus*	Common Wood Pigeon	5726	10.85%	
Passeriformes	*Passer italiae*	Italian Sparrow	4318	8.18%	
Passeriformes	*Sturnus vulgaris*	Common Starling	4223	8.00%	
Passeriformes	*Hirundo rustica*	Barn Swallow	3098	5.87%	
Columbiformes	*Streptopelia decaocto*	Eurasian Collared Dove	2514	4.76%	
Anseriformes	*Anas platyrhynchos*	Mallard	2066	3.91%	Yes
Passeriformes	*Fringilla coelebs*	Common Chaffinch	1525	2.89%	
Passeriformes	*Turdus merula*	Common Blackbird	1521	2.88%	
Charadriiformes	*Chroicocephalus ridibundus*	Black-headed Gull	1499	2.84%	Yes
Passeriformes	*Pica pica*	Eurasian Magpie	1371	2.60%	Yes
Charadriiformes	*Larus michahellis*	Yellow-legged Gull	1200	2.27%	Yes
Passeriformes	*Carduelis carduelis*	European Goldfinch	1144	2.17%	
Passeriformes	*Carduelis chloris*	European Greenfinch	1053	1.99%	
Passeriformes	*Sylvia atricapilla*	Eurasian Blackcap	794	1.50%	
Gruiformes	*Gallinula chloropus*	Common Moorhen	760	1.44%	
Passeriformes	*Anthus pratensis*	Meadow Pipit	724	1.37%	
Passeriformes	*Corvus cornix*	Hooded Crow	721	1.37%	
Passeriformes	*Aegithalos caudatus*	Long-tailed Tit	712	1.35%	
Suliformes	*Phalacrocorax carbo*	Great Cormorant	667	1.26%	Yes
Galliformes	*Phasianus colchicus*	Common Pheasant	647	1.23%	
Passeriformes	*Erithacus rubecula*	European Robin	629	1.19%	
Passeriformes	*Parus major*	Great Tit	585	1.11%	
Pelecaniformes	*Bubulcus ibis*	Cattle Egret	579	1.10%	
Passeriformes	*Motacilla flava*	Western Yellow Wagtail	558	1.06%	
Passeriformes	*Motacilla alba*	White Wagtail	478	0.91%	
Passeriformes	*Passer montanus*	Eurasian Tree Sparrow	399	0.76%	
Passeriformes	*Merops apiaster*	European Bee-eater	372	0.70%	
Pelecaniformes	*Ardea cinerea*	Grey Heron	339	0.64%	Yes
Pelecaniformes	*Egretta garzetta*	Little Egret	334	0.63%	Yes
Passeriformes	*Luscinia megarhynchos*	Common Nightingale	311	0.59%	
Passeriformes	*Garrulus glandarius*	Eurasian Jay	303	0.57%	

**Order**	**Scientific name**	**Common name**	**No. birds**	**Percentage**	**Target AI**
Passeriformes	*Troglodytes troglodytes*	Eurasian Wren	291	0.55%	
Apodiformes	*Apus apus*	Common Swift	265	0.50%	
Piciformes	*Dendrocopos major*	Great Spotted Woodpecker	248	0.47%	
Passeriformes	*Corvus monedula*	Eurasian Jackdaw	229	0.43%	
Piciformes	*Picus viridis*	European Green Woodpecker	218	0.41%	
Passeriformes	*Cettia cetti*	Cetti's Warbler	214	0.41%	
Passeriformes	*Phylloscopus collybita*	Common Chiffchaff	205	0.39%	
Passeriformes	*Delichon urbicum*	Common House Martin	188	0.36%	
Charadriiformes	*Vanellus vanellus*	Northern Lapwing	177	0.34%	
Passeriformes	*Oriolus oriolus*	Eurasian Golden Oriole	176	0.33%	
Passeriformes	*Cisticola juncidis*	Zitting Cisticola	172	0.33%	
Passeriformes	*Serinus serinus*	European Serin	153	0.29%	
Anseriformes	*Anas crecca*	Eurasian Teal	149	0.28%	Yes
Passeriformes	*Regulus regulus*	Goldcrest	146	0.28%	
Suliformes	*Phalacrocorax pygmeus*	Pygmy Cormorant	142	0.27%	
Accipitriformes	*Buteo buteo*	Common Buzzard	141	0.27%	Yes
Columbiformes	*Streptopelia turtur*	European Turtle Dove	133	0.25%	
Falconiformes	*Falco tinnunculus*	Common Kestrel	131	0.25%	
Anseriformes	*Aythya ferina*	Common Pochard	124	0.23%	Yes
Passeriformes	*Alauda arvensis*	Skylark	121	0.23%	
Gruiformes	*Fulica atra*	Eurasian Coot	118	0.22%	
Podicipediformes	*Tachybaptus ruficollis*	Little Grebe	114	0.22%	Yes
Pelecaniformes	*Threskiornis aethiopicus*	African Sacred Ibis	100	0.19%	
Pelecaniformes	*Casmerodius albus*	Great Egret	90	0.17%	Yes
Passeriformes	*Ptyonoprogne rupestris*	Eurasian Crag Martin	88	0.17%	
Passeriformes	*Anthus spinoletta*	Water Pipit	62	0.12%	
Podicipediformes	*Podiceps cristatus*	Great Crested Grebe	62	0.12%	Yes
Passeriformes	*Saxicola torquatus*	Common Stonechat	60	0.11%	
Charadriiformes	*Tringa totanus*	Common Redshank	55	0.10%	
Passeriformes	*Emberiza calandra*	Corn Bunting	53	0.10%	
Passeriformes	*Carduelis spinus*	Eurasian Siskin	51	0.10%	
Passeriformes	*Sylvia borin*	Garden Warbler	49	0.09%	
Passeriformes	*Acrocephalus scirpaceus*	Eurasian Reed Warbler	46	0.09%	
Anseriformes	*Anser anser*	Greylag Goose	46	0.09%	Yes

Order	Scientific name	Common name	No. birds	Percentage	Target AI
Phoenicopteriformes	*Phoenicopterus roseus*	Greater Flamingo	46	0.09%	
Charadriiformes	*Sterna hirundo*	Common Tern	46	0.09%	
Coraciiformes	*Alcedo atthis*	Common Kingfisher	44	0.08%	
Passeriformes	*Emberiza schoeniclus*	Reed Bunting	44	0.08%	
Anseriformes	*Tadorna tadorna*	Common Shelduck	44	0.08%	Yes
Charadriiformes	*Actitis hypoleucos*	Common Sandpiper	43	0.08%	
Passeriformes	*Phoenicurus ochruros*	Black Redstart	39	0.07%	
Passeriformes	*Cyanistes caeruleus*	Eurasian Blue Tit	38	0.07%	
Pelecaniformes	*Ardea purpurea*	Purple Heron	36	0.07%	
Anseriformes	*Aythya nyroca*	Ferruginous Duck	36	0.07%	
Passeriformes	*Muscicapa striata*	Spotted Flycatcher	35	0.07%	
Passeriformes	*Prunella modularis*	Dunnock	32	0.06%	
Charadriiformes	*Larus melanocephalus*	Mediterranean Gull	31	0.06%	
Anseriformes	*Aythya fuligula*	Tufted Duck	30	0.06%	
Charadriiformes	*Gelochelidon nilotica*	Gull-billed Tern	29	0.05%	
Passeriformes	*Acrocephalus arundinaceus*	Great Reed Warbler	24	0.05%	
Anseriformes	*Anas querquedula*	Garganey	24	0.05%	
Cuculiformes	*Cuculus canorus*	Common Cuckoo	23	0.04%	
Charadriiformes	*Gallinago gallinago*	Common Snipe	23	0.04%	
Passeriformes	*Anthus campestris*	Tawny Pipit	19	0.04%	
Pelecaniformes	*Nycticorax nycticorax*	Black-crowned Night Heron	19	0.04%	
Accipitriformes	*Circus aeruginosus*	Western Marsh Harrier	18	0.03%	
Passeriformes	*Phylloscopus trochilus*	Willow Warbler	18	0.03%	
Charadriiformes	*Tringa glareola*	Wood Sandpiper	18	0.03%	
Passeriformes	*Hippolais polyglotta*	Melodious Warbler	17	0.03%	
Charadriiformes	*Tringa nebularia*	Common Greenshank	17	0.03%	
Accipitriformes	*Circus pygargus*	Montagu's Harrier	16	0.03%	
Accipitriformes	*Circus cyaneus*	Northern Harrier	15	0.03%	
Charadriiformes	*Tringa ochropus*	Green Sandpiper	15	0.03%	Yes
Passeriformes	*Phoenicurus phoenicurus*	Common Redstart	14	0.03%	
Charadriiformes	*Sterna sandvicensis*	Sandwich Tern	14	0.03%	
Passeriformes	*Turdus pilaris*	Fieldfare	13	0.02%	Yes
Passeriformes	*Acrocephalus palustris*	Marsh Warbler	12	0.02%	
Passeriformes	*Riparia riparia*	Sand Martin	12	0.02%	
Pelecaniformes	*Ardeola ralloides*	Squacco Heron	11	0.02%	

Order	Scientific name	Common name	No. birds	Percentage	Target AI
Bucerotiformes	*Upupa epops*	Eurasian Hoopoe	11	0.02%	
Passeriformes	*Phylloscopus sibilatrix*	Wood	10	0.02%	
Strigiformes	*Athene noctua*	Little Owl	9	0.02%	
Charadriiformes	*Haematopus ostralegus*	Eurasian Oystercatcher	9	0.02%	
Podicipediformes	*Podiceps nigricollis*	Black-necked Grebe	9	0.02%	Yes
Galliformes	*Coturnix coturnix*	Common Quail	8	0.02%	
Accipitriformes	*Accipiter nisus*	Eurasian Sparrowhawk	7	0.01%	
Passeriformes	*Motacilla cinerea*	Grey Wagtail	7	0.01%	
Passeriformes	*Oenanthe oenanthe*	Northern Wheatear	7	0.01%	
Gruiformes	*Rallus aquaticus*	Water Rail	7	0.01%	
Passeriformes	*Remiz pendulinus*	Eurasian Penduline Tit	7	0.01%	
Passeriformes	*Turdus philomelos*	Song Thrush	7	0.01%	
Passeriformes	*Carduelis cannabina*	Eurasian Linnet	6	0.01%	
Anseriformes	*Cygnus olor*	Mute Swan	6	0.01%	Yes
Falconiformes	*Falco subbuteo*	Eurasian Hobby	6	0.01%	
Passeriformes	*Lanius collurio*	Red-backed Shrike	6	0.01%	
Galliformes	*Perdix perdix*	Grey Partridge	6	0.01%	
Passeriformes	*Galerida cristata*	Crested Lark	5	0.01%	
Pelecaniformes	*Ixobrychus minutus*	Little Bittern	5	0.01%	
Piciformes	*Jynx torquilla*	Eurasian Wryneck	5	0.01%	
Pelecaniformes	*Plegadis falcinellus*	Glossy Ibis	5	0.01%	
Passeriformes	*Sylvia communis*	Common Whitethroat	5	0.01%	
Anseriformes	*Anas acuta*	Northern Pintail	4	0.01%	Yes
Accipitriformes	*Falco peregrinus*	Peregrine Falcon	4	0.01%	Yes
Gruiformes	*Grus grus*	Common Crane	4	0.01%	
Charadriiformes	*Larus canus*	Common Gull	4	0.01%	Yes
Passeriformes	*Saxicola rubetra*	Whinchat	4	0.01%	
Passeriformes	*Fringilla montifringilla*	Brambling	3	0.01%	
Passeriformes	*Turdus viscivorus*	Mistle Thrush	3	0.01%	
Charadriiformes	*Chlidonias leucopterus*	White-winged Tern	2	0.004%	
Coraciiformes	*Coracias garrulus*	European Roller	2	0.004%	
Passeriformes	*Emberiza citrinella*	Yellowhammer	2	0.004%	
Charadriiformes	*Numenius arquata*	Eurasian Curlew	2	0.004%	
Strigiformes	*Otus scops*	Eurasian Scops Owl	2	0.004%	
Passeriformes	*Periparus ater*	Coal Tit	2	0.004%	
Accipitriformes	*Pernis apivorus*	European Honey	2	0.004%	

**Order**	**Scientific name**	**Common name**	**No. birds**	**Percentage**	**Target AI**
		Buzzard			
Passeriformes	*Corvus corone*	Carrion Crow	1	0.002%	
Passeriformes	*Emberiza cia*	Rock Bunting	1	0.002%	
Passeriformes	*Ficedula hypoleuca*	Pied Flycatcher	1	0.002%	
Charadriiformes	*Hydroprogne caspia*	Caspian Tern	1	0.002%	
Psittaciformes	*Melopsittacus undulatus*	Budgerigar	1	0.002%	
Psittaciformes	*Psittacula krameri*	Rose-ringed Parakeet	1	0.002%	
Passeriformes	*Pyrrhula pyrrhula*	Eurasian Bullfinch	1	0.002%	
Passeriformes	*Sitta europaea*	Eurasian Nuthatch	1	0.002%	
Strigiformes	*Tyto alba*	Barn Owl	1	0.002%	
**Total**			**52794**	**100%**	**21**

Table S2. List of target species for passive surveillance of Avian Influenza in wild birds established by EFSA in 2017 (adapted from More et al., 2017).

**Family**	**Subfamily, tribe, or genus**	**Species**	**% positive (no.positive/no. tested)**
Ducks, geese, and swans (Anatidae)	Diving ducks (Aythyini)	Tufted duck (*Aythya fuligula*)	33.4% (338/1011)
		Greater scaup (*Aythya marila*)	12.7% (9/71)
		Common pochard (*Aythya ferina*)	11.4% (26/228)
		Red-crested pochard (*Netta rufina*)	0.9% (1/112)
	Dabbling ducks (Anatinae)	Northern pintail (*Anas acuta*)	5.4% (3/56)
		Eurasian wigeon (*Anas penelope*)	3.7% (8/219)
		Gadwall (*Anas strepera*)	1.7% (3/179)
		Mallard (*Anas platyrhynchos*)	0.5% (96/20672)
		Eurasian teal (*Anas crecca*)	0.4% (5/1145)
	Sea ducks (Mergini)	Goosander (*Mergus merganser*)	6.4% (7/109)
		Common goldeneye (*Bucephala clangula*)	5.7% (3/53)
		Smew (*Mergus albellus*)	5.0% (1/20)
		Common eider (*Somateria mollissima*)	1.3% (3/228)
	Shelducks and sheldgeese (Tadorninae)	Common shelduck (*Tadorna tadorna*)	0.5% (1/218)
		Egyptian goose (*Alopochen aegyptiacus*)	0.4% (1/234)
	True geese (Anser, Branta, Chen)	Lesser white-fronted goose (*Anser erythropus*)	13% (3/23)
		Greylag goose (*Anser anser*)	3.5% (68/1968)
		Taiga bean Goose (*Anser fabalis*)	2.8% (4/143)
		Canada goose (*Branta canadensis*)	1.8% (19/1061)

**Family**	**Subfamily, tribe, or genus**	**Species**	**% positive (no.positive/no. tested)**
		Pink-footed goose (*Anser brachyrhynchus*)	1.3% (1/75)
		Brant goose (*Branta bernicla*)	1.2% (1/84)
		Greater white-fronted goose (*Anser albifrons*)	0.6% (2/350)
	Swans (Cygnus)	Black swan (*Cygnus atratus*)	9.5% (6/63)
		Whooper swan (*Cygnus cygnus*)	9.3% (169/1818)
		Mute swan (*Cygnus olor*)	7.6% (931/12268)
Grebes (Podicipedidae)		Black-necked grebe (*Podiceps nigricollis*)	79.9% (246/308)
		Great crested grebe (*Podiceps cristatus*)	8.5% (50/588)
		Little grebe (*Tachybaptus ruficollis*)	7.8% (6/77)
Storks (Ciconiidae)		White stork (*Ciconia ciconia*)	0.5% (5/911)
Herons (Ardeidae)		Eurasian bittern (*Botaurus stellaris*)	2.9% (1/35)
		Little egret (*Egretta garzetta*)	2.9% (2/69)
		Great white egret (*Egretta alba*)	0.9% (4/441)
		Grey heron (*Ardea cinerea*)	0.8% (40/5093)
Pelicans (Pelecanidae)		Dalmatian pelican (*Pelecanus crispus*)	27.5% (11/40)
		Great white pelican (*Pelecanus onocrotalus*)	9.5% (2/21)
Cormorants and shags (Phalacrocoracidae)		Great cormorant (*Phalacrocorax carbo*)	0.6% (12/2090)
Raptors (Accipitridae, Falconidae, Strigidae)		White-tailed eagle (*Haliaeetus albicilla*)	6.6% (28/426)
		Rough-legged buzzard (*Buteo lagopus*)	3.7% (1/27)
		Common buzzard (*Buteo buteo*)	1.1% (72/6307)
		Peregrine falcon (*Falco peregrinus*)	3.4% (10/297)
		Northern goshawk (*Accipiter gentilis*)	1.3% (8/616)
		Eurasian eagle-owl (*Bubo bubo*)	0.9% (3/340)
Coots, crakes, and rails (Rallidae)		Western swamphen (*Porphyrio porphyrio*)	6.7% (1/15)
Sandpipers (Scolopacidae)[(b)]		Green sandpiper (*Tringa ochropus*)	33.3% (1/3)
Gulls, terns, and allies (Laridae)		Great black-backed gull (*Larus marinus*)	13.8% (22/159)
		European herring gull (*Larus argentatus*)[(a)]	3.1% (66/2135)
		Mew gull (*Larus canus*)	0.8 (4/481)
		Black-headed gull (*Chroicocephalus ridibundus*)	0.7% (30/4075)
Corvids (Corvidae)		Eurasian magpie (*Pica pica*)	0.6% (7/1232)
Thrushes (Turdidae)		Fieldfare (*Turdus pilaris*)	0.5% (1/192)

[(a)] This does not include the Caspian gull (*Larus cachinnans*) or the yellow-legged gull (*Larus michahellis*), which are considered separate species. [(b)] Another wader, Numenius species was not included in this list because it was not identified to species. However, in the EU, the two most likely Numenius species are the Eurasian curlew (*N. arquata*) and the whimbrel (*N. phaeopus*).

Table S3. Pairwise Wilcoxon test calculated on the distributions of wild birds encountered in ornithological transects. Correction method: Benjamini-Hochberg. Significant p-values (<0.05) are highlighted in green.

	BO-01	FE-01	FE-02	MN-01	MN-02	RO-01	VE-01	VE-02	VR-01
**FE-01**	0.375								
**FE-02**	0.180	0.153							
**MN-01**	0.289	0.146	0.689						
**MN-02**	0.002	0.010	0.119	0.023					
**RO-01**	0.023	0.025	0.105	0.075	0.703				
**VE-01**	9.975	0.0001	0.0003	0.0001	0.005	0.068			
**VE-02**	0.153	0.077	0.289	0.344	0.812	0.657	0.050		
**VR-01**	0.014	0.014	0.223	0.102	0.696	0.718	0.020	0.657	
**VR-02**	0.000	0.001	0.011	0.002	0.153	0.289	0.223	0.153	0.171

Table S4. Pairwise Wilcoxon test calculated between the distributions of at the Chao1 richness indices for the three clusters. Correction method: Benjamini-Hochberg. Significant p-values (<0.05) are highlighted in green.

	Cluster 1	Cluster 2
**Cluster 2**	0.0002	
**Cluster 3**	0.0002	0.023

Table S5. Pairwise Wilcoxon test calculated between the distributions of Shannon diversity indices for the three clusters. Correction method: Benjamini-Hochberg. Significant p-values (<0.05) are highlighted in green.

	Cluster 1	Cluster 2
**Cluster 2**	0.0003	
**Cluster 3**	0.285	0.0001

Table S6. Pairwise Wilcoxon test calculated between the distributions of Pielou's eveness diversity indices for the three clusters. Correction method: Benjamini-Hochberg. Significant p-values (<0.05) are highlighted in green.

	Cluster 1	Cluster 2
**Cluster 2**	0.016	
**Cluster 3**	0.012	0.0004

Table S7. Installation and removal dates for the camera-traps in each farm.

Camera traps location	Installation Date	Removal Date
BO-01-A	25/01/2019	31/12/2019
BO-01-B	25/01/2019	31/12/2019
FE-01-A	07/01/2019	31/12/2019
FE-01-B	07/01/2019	31/12/2019
FE-02-A	16/01/2019	31/12/2019
FE-02-B	16/01/2019	31/12/2019
MN-01-A	28/01/2019	31/12/2019
MN-01-B	28/01/2019	31/12/2019
MN-02-A	11/01/2019	31/12/2019
MN-02-B	11/01/2019	31/12/2019

Camera traps location	Installation Date	Removal Date
RO-01-A	01/01/2019	31/12/2019
RO-01-B	01/01/2019	31/12/2019
VE-01-A	08/02/2019	31/12/2019
VE-01-B	08/02/2019	31/12/2019
VE-02-A	15/01/2019	31/12/2019
VE-02-B	15/01/2019	31/12/2019
VR-01-A	03/01/2019	31/12/2019
VR-01-B	03/01/2019	31/12/2019
VR-02-A	21/01/2019	31/12/2019
VR-02-B	21/01/2019	31/12/2019

The total number of camera trap days was 6988, while the total daily events per farms were 1936, equal to 2055 daily events per camera trap.
